# What predicts self-efficacy? Understanding the role of sociodemographic, behavioural and parental factors on condom use self-efficacy among university students in Nigeria

**DOI:** 10.1371/journal.pone.0221804

**Published:** 2019-08-28

**Authors:** Anthony Idowu Ajayi, Emmanuel Olawale Olamijuwon

**Affiliations:** 1 Population Dynamics and Sexual and Reproductive Health Unit, African Population and Health Research Centre, APHRC Campus, Manga Close, Nairobi, Kenya; 2 Department of Statistics and Demography, Faculty of Social Science, University of Eswatini (formerly Swaziland), Kwaluseni, Eswatini; 3 Demography and Population Studies Programme, Schools of Public Health and Social Science, University of the Witwatersrand, Johannesburg, South Africa; University of California Los Angeles, UNITED STATES

## Abstract

**Background:**

Risky sexual behaviours are not uncommon among young adults particularly those in the higher levels of education. It is known that higher self-efficacy could contribute to better sexual and reproductive health outcomes including the use of condoms. However, there is limited research on the role of socio-demographic, behavioural and parental factors as predictors of condom-use self-efficacy. As a result, this exploratory study was designed to assess the predictors of self-efficacy for condom use among university students in Nigeria

**Methods:**

A cross-sectional survey was conducted with 755 university students in Nigeria from February to April 2018. Self-efficacy for condom use was assessed by combining responses to 11-items measures of condom self-efficacy drawn from the work of Barkley and colleagues. We fitted a structural equation model to identify the pathways through which socio-demographic, behavioural and parental factors predict two constructs of condom-use self-efficacy (self-efficacy for condom purchase and use and partner communication self-efficacy) in the sample.

**Results:**

Demographic factors such as age *(β = -0*.*29*, *p<0*.*05)* and sex *(β = 0*.*42*, *p<0*.*05)*, as well as ratings on religious importance *(β = -0*.*08*, *p<0*.*05)* were directly associated with self-efficacy for condom purchase and use. These factors showed significantly mediated effects through sexual experience which also had a direct positive relationship *(β = 0*.*73*, *p<0*.*05)* with self-efficacy for condom purchase and use. The receipt of parental support, on the other hand, was directly associated with higher partner communication efficacy for condom use *(β = 0*.*07*, *p<0*.*05)*. We found no evidence that the level of partner communication efficacy was directly associated with any of the behavioural, demographic or parental factors.

**Conclusion:**

The findings of this study affirm that sex, or age or having higher ratings on religious importance alone does not increases self-efficacy but also exposure to sexual activity through which these factors affect self-efficacy for condom purchase and use. These findings also highlight the need to address and strengthen condom use self-efficacy among young adults, particularly the sexually inexperienced, highly religious and young adults with limited support from their parent.

## Introduction

### Background

Condom use, especially among adolescents and young adults, is crucial for the prevention of human immunodeficiency virus (HIV) and other sexually transmitted diseases (STDs) transmission [1]. Although there has been a rapid increase in sexual health promotions targeting young adults in sub-Saharan Africa [2–4], the proportion of youth still engaging in risky sexual behaviours remains staggering [5, 6]. For instance, a study reported that over 60% of sexually active unmarried female university students in two Nigerian universities engaged in unprotected sex [7]. Consequently, a high prevalence of unplanned pregnancy, unsafe abortion, STIs, and HIV has been reported among young adults in sub-Saharan Africa [8, 9]. Risky sexual behaviour could jeopardise the sexual and reproductive health and wellbeing of young adults.

Self-efficacy is an individual’s confidence in his/her ability to exercise control over his/her behaviour and environment, [10–12]. According to Badura [13, 14], self-efficacy is developed through personal experience, social learning and social persuasion and has since been applied in several behavioural change research and practice [15–18]. Condom self-efficacy refers to an individual’s confidence in his/her ability to purchase condoms, negotiate the use of condoms with partners, and use condoms during sexual intercourse [19–21]. Studies have consistently shown that condom self-efficacy is an important determinant of condom use and intention to use condoms [22–24]. It is clear that people who have a high sense of condom self-efficacy are likely to use condoms, whereas those who have self- doubts about their ability to use condoms are less likely to do so [24]. Studies have also shown that counselling young adults on condom self-efficacy promotes condom use and leads to HIV risk reduction [25, 26]. Thus, increasing self-efficacy remains a crucial recommendation for improving condom use among young adults [22–24].

Although the relationship between condom self-efficacy and condom use is well established, only a few studies have examined the factors associated with condom use self-efficacy among young adults [27, 28]. The available studies have shown gender differences and ethnic variations in condom use self-efficacy [27, 28]. These studies reported varying results and mostly conducted in developed countries. The available evidence from South Africa has also examined the gender differences in the predictor of condom use self-efficacy, but attention was given to behavioural factors evidenced in the knowledge of HIV prevention, HIV communication and ambitions [27].

Despite the low rate of condom use [7], a high prevalence of unplanned pregnancy [8] and unsafe abortions [29, 30] among young adults in Nigeria, very little studies have attempted to improve our understanding of the socio-demographic, behavioural and parental predictors of condom use self-efficacy in an African sample as Nigeria. As a result, this study focuses on identifying socio-demographic, behavioural and parental factors associated with condom use self-efficacy among young adults in Nigeria. Profiling of individuals with low condom self-efficacy could help to design bespoke intervention that meets the need of the most at-risk group.

### Literature review and theoretical framework

**Fig 1:**
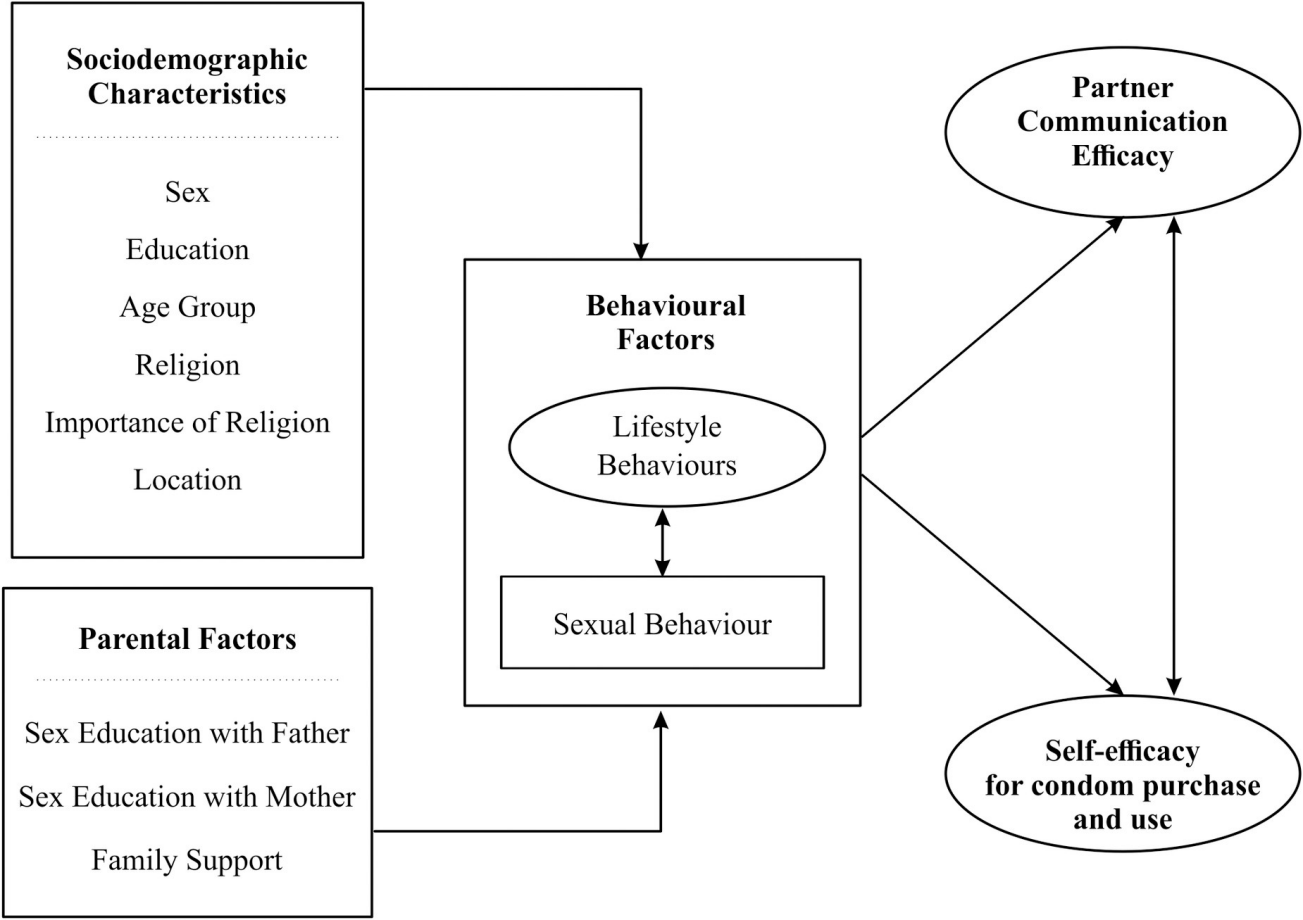
Proposed model for understanding the predictors of condom use self-efficacy among university students in Nigeria.

We build upon what is known in the body of knowledge and the existing theoretical models in linking socio-demographic, behavioural and parental factors with condom use self-efficacy among young adults in Nigeria. We propose a multidimensional model illustrated in Fig 1 to argue that socio-demographic, and parental factors will affect condom use self-efficacy directly and through the behavioural factors.

We hypothesize that behavioural factors such as sexual experience, alcohol use, drug use and tobacco smoking could influence condom self-efficacy directly. This hypothesis is premised on Badura’s theory of self-efficacy [13, 14], which asserts that self-efficacy is developed through personal experience, social learning and social persuasion. Sexually experienced young adults may be more confident in their ability to access and negotiate condoms use than those who have never had sex. Through personal experience and performance accomplishment attained during previous sexual encounters, young adults could have strengthened their self-efficacy of condom use [10–12]. Given this premise, we propose that sexual experience is positively associated with higher self-efficacy score. Also, studies have shown that substance use is associated with risky sexual behaviour [31–33]. While alcohol and drug use could boost confidence, it could also limit an individual’s agency to negotiate condom use. A previous study has shown that alcohol intoxication decreases intentions both to negotiate condom use and to use condoms in the future [34]. It is, therefore, essential to explore how substance use in general impact condom self-efficacy.

We also hypothesize that socio-demographic factors such as age, sex and level of education and religiosity may affect condom use self-efficacy. Previous studies have shown that males tend to have higher condom self-efficacy score compared to female [27, 28]. Likewise, health behaviour theories often highlight the importance of individual-level factors such as age, sex, location, religion and income on sexual and reproductive health outcomes [17, 35, 36]. Similarly, studies have shown that religion and religiosity have a strong positive influence on sexual behaviour and experience of adolescents and young adults [37, 38]. Highly religious young adults in Nigeria are more likely to abstain from sexual activity than less-religious young adults [38]. While religious teachings emphasise sexual abstinence and preclude the use of contraceptives, we surmised that these teachings could inhibit the condom self-efficacy of young adults. Moreover, given the expected pattern of relationship between sexual experience and condom use self-efficacy as well as religiosity and sexual experience, we expect that young adult’s level of religiosity would negatively affect condom use self-efficacy.

Finally, our study hypothesizes that parental factors would positively affect condom use self-efficacy. We assert that parental communication, particularly on topics relating to sexual and reproductive health, would positively influence condom self-efficacy of adolescents and young adults both directly and indirectly. Although the effect of parental factors on condom self-efficacy has rarely been investigated, the results of previous studies on the role of parental communication on sex and contraceptive at least lay credence to our assumption [39–41]. Previous studies have also highlighted the significance of parental factors in protective sexual behaviour of adolescents and young adults [39–41]. As a result, we expect that young people with more support from their parents and communicate with their parents on topics relating to sexual and reproductive health may have higher condom self-efficacy than those who with limited support from home and communicate less with their parents.

We tested all our assertions in this study by fitting a robust structural equation model on cross-sectional data obtained from students selected from two Nigeria universities.

## Methodology

### Data

We conducted a cross-sectional survey among adolescents and young adults in two universities in Nigeria from February to April 2018 using an interviewer-administered questionnaire. We focus on university students because unlike their counterparts in high school or who stopped schooling at the high school level; they have a significantly high risk of a premarital and unwanted pregnancy due to the long waiting time they are exposed to from sexual initiation to marriage. Previous studies have also shown that high-risk sexual behaviour is common on Nigerian campuses [7, 42]. The heightened need for condom use among this group of young adults and other factors underscores the need for understanding the predictors of condom use self-efficacy in this population.

The University of Ilorin and the Nasarawa State University, located both in North Central Nigeria, were selected based on the known prevalence of HIV in the states in which they are situated. Nasarawa State is generally known for the high HIV prevalence while the prevalence of HIV is much lower in Ilorin [43]. Before the survey, a pilot study was conducted in another university and among 20 participants who were not included in the main study. Feedback from these participants was used to improve the questionnaire. Field workers had prior data collection experience and were specifically trained on the project objectives, research ethics and sampling strategy.

Ethical approval for the study was obtained from the University of Fort Hare, South Africa and the Ondo State Ministry of Health Ethical Review Committees. The study was conducted in accordance with all relevant ethical guidelines and regulations. Eligible participants were given a copy of the informed consent form, which outlined the objectives of the study, confidentiality measures as well as the details of the principal investigator. Participants were also informed that participation in the study was completely voluntary and that they were free not to answer any question and stop the survey at any point. For students under age 18 years, we provided them with an additional parental/guardian consent form to complete. The students were given a week to obtain their parent/guardian’s consent to participate in the study. About 56 under-18 students who were able to obtain their parent or guardian’s consent within the period and assented to participate were included in the study.

The survey collected rich information on family support, religiosity and sexual health knowledge, and behaviours among adolescents and young adults in the two universities. Information on HIV knowledge and attitudes were also collected. The recency of the survey combined with the rich information collected makes it a very valuable resource for studying the recent level of condom use self-efficacy among university students in Nigeria.

### Study participants

A stratified sampling technique was employed among male/female strata in the two universities. A sample of 400 (200 males and 200 females) participants at a confidence level of 95%, a 5% precision level, and adjusted for missing responses, was required in each university. The estimated sample size was premised on the fact that the student population in both universities were about 45,000 students. Similarly, the proportions of male to female students at both universities were nearly equal. Further details of the sampling strategy have been presented elsewhere [44].

With a response rate of about 98%, the full data comprised of about 784 students in both universities. We further excluded about 4% of the sample with incomplete information on basic demographic characteristics as well as the key measures of condom self-efficacy. The final analytic sample in our study was therefore 755 young adults who were currently enrolled in the two universities.

### Measures

We adopted a latent variable structural equation model with two primary endogenous variables (condom use self-efficacy) and two other secondary endogenous variables (behavioural factors) and multiple exogenous variables (socio-demographic and parental factors).

#### Primary endogenous measures

The primary endogenous variable for this study is condom efficacy, which was drawn from the work of Barkley and colleagues [45]. The construct contains information on the self-rated ability of young adults to access and use a condom at any sexual encounter. The measure comprised of questions such as *“I would not feel confident suggesting using condoms with a new partner because they would be afraid that he or she would think they have a sexually transmitted disease”*. In their analysis, Barkley and colleagues adapted an initial 28 item condom self-efficacy construct to develop a three-factor construct of condom self-efficacy comprising of assertiveness, partner’s reaction and STDs. We further added three items suitable to our context to the final 10-item construct of condom self-efficacy in Barkley and colleagues’ study [45]. However, during analysis, we excluded two-items that had lower loadings on either construct of condom self-efficacy in our study.

The response categories for each of the measure were rated on a 3-point Likert scale with categories 1 (Disagree), 2 (Neutral) and 3 (Agree). Responses to reverse questions were reverse coded so that higher values indicated a desirable response and lower values indicated an undesirable response. An exploratory factor analysis was used to identify two latent constructs comprising of self-efficacy for condom purchase and use and partner communication self-efficacy. We use the term self-efficacy for condom purchase and use to refer to the ability of young adults to be able to successfully use, access or purchase a condom while partner communication self-efficacy referred to the ability of young adults to be able to successfully communicate or negotiate condom use with their sexual partner(s). Cronbach alpha reliability estimate for the two latent constructs provided evidence in support of a strong and acceptable internal reliability (α = 0.867 for self-efficacy for condom purchase and use, and α = 0.866 for partner communication self-efficacy).

#### Secondary endogenous measures

The secondary endogenous variable for this study comprised of variables that appear as dependent variables in at least one of the equations. This includes behavioural factors such as sexual experience and lifestyle behaviours. *Sexual experience* was a binary response variable that was assessed by asking if the participants have ever had sex or not. *Lifestyle behaviours*, on the other hand, was examined as a latent construct consisting of young adults’ recent consumption of alcohol, drug use or cigarettes. Study participants were asked if they have ever consumed alcohol and if yes if they still do *(in the last month)*. The response categories for each of the measure were rated on 3-point Likert scale with categories 1 “Never did” 2 “No longer do” and 3 “currently do”. An exploratory factor analysis was used to identify two latent constructs comprising of self-efficacy for condom purchase and use and partner communication self-efficacy. Cronbach alpha reliability estimate for this latent construct of lifestyle behaviours provided evidence in support of a strong and acceptable internal reliability (α = 0.806).

#### Exogenous measures

The exogenous variables in this study comprise variables that were considered as an independent variable in all the equations. This comprised of the participants’ socio-demographic factors (age, sex, level of education, the importance of religion) and the parental factors (sex education with father, sex education with mother, and parental support).

Information on age was assessed by asking the students to indicate which of the age groups was most applicable. The categories included “adolescents (16–19 years)”, and “young adults (20–34 years)”. The students were also asked to indicate the sex that they self-identify with. This includes male/female. Although our study was among university students, we included an indicator variable of their current level of education in the university as an assessment of their level of exposure and knowledge. The response variable was categorized as “100 L” for participants who are in their first year, “200 L” for those in the second year, “300 L” for those in the third year and “400+ L” for those in the fourth or more years. The participants were also asked to rate the level of importance of religion in their lives. The response variable was categorized as “Not important”, “Moderately important” and “very important”.

Similarly, information on the level of sex education with father or mother was assessed from the combination of questions on whether study participants have ever discussed sex-related matters with their mother and if yes, how frequent they do so. Responses to the questions were combined and measured on binary scale with categories such as “often/occasionally” for those who reported that they have once and often/occasionally discuss sex-related matters with their mother, “never” for those who reported that they have never discussed sex-related matters with their mother or not currently not living with their mother. Similar measurement approach was adopted for sex education with father. Finally, participants were asked if they receive any financial or emotional support from their family. This variable was examined as a binary variable with categories “receive no support” and “receive support”.

### Analytical approach

The data were analysed using Stata/MP (v. 14.0). Descriptive statistics comprising of frequency and percentage distributions were used to describe the profile of adolescents and young adults in the sample in relation to their demographics. Pearson’s *r* correlation coefficient was used to determine the multicollinearity between the variables. An exploratory factor analysis (EFA) was used to determine the most plausible factor structure for the dimensions of condom use self-efficacy and the best performing items to retain. We retained factors based on their interpretability or the extent to which items in the same factor are related to another (i.e., a dimension of religiosity), the significance of factor loadings and goodness of fit.

We further employed a two-stage latent variable structural equation model, which included both measurement and structural portions. First, a three-factor confirmatory factor analysis examined the appropriateness and generalizability of the latent constructs of lifestyle behaviours, self-efficacy for condom purchase and use and partner communication self-efficacy. This is premised on the fact that the underlying unmeasured variables can be identified by the shared variance of the observed variables in the data. Model fit for the CFA was assessed through the fit statistics and the statistical significance of paths. We considered a root-mean-square error of approximation (RMSEA) that is 0.05 or less; a standardized root mean square residual (SRMR) that is less than 0.08; a Tucker-Lewis index (TLI) and comparative fit index (CFI) that are 0.90 or greater as indicators of a good model fit [46–49]. Convergent validity, discriminant validity and construct reliability were also examined to assess the quality of the measurement model. Both the convergent validity and the discriminant validity were estimated using the *relicoef* package while the composite construct reliability was estimated using the *condisc* package in Stata [50, 51]

To answer our main research question of what factors, determine condom use self-efficacy among adolescents and young adults in Nigeria, we further tested the structural relationships among sociodemographic factors, parental factors, behavioural factors and condom use self-efficacy. Our model analysed four structural equations simultaneously for the five endogenous variables in the model. Given that participants in the study were recruited from different sites (two universities), we also included an indicator of the recruitment site in the equations. Because our measures are ordinal and binary, we use the asymptotic distribution-free estimator which has been shown to produce consistent parameter estimates, correct standard errors, and accurate fit statistics for categorical indicator variables [46, 52]. The errors of condom use self-efficacy were all co-varying due to the possible relatedness among the unobserved aspects of these constructs. Modification indices were also reviewed to assess possible correlated errors. The fitness of the final model was also assessed based on standard fit statistics such as RMSEA < 0.05; TLI ≥ 0.90; CFI ≥ 0.90; and SRMR < 0.80 [46–49, 53].

## Results

### Descriptive profile of the sample

Table 1 presents a summary description of young adults in the sample. About 27% of young adults in the sample are adolescents. Just over half (51%) of the participants are males and slightly more than one-quarter of the sample was in the fourth year of study. Most of the participants (92%) also reported receiving support from families at home. More than three-quarters of the young adults reported that religious activities were very important to them. More than three-quarters of the young adults have had sex and only about 19% reported to have never had sex. Only about 17% of the young adults reported to occasionally or often discuss sex with their father. On the other hand, about 47% of young adults reported to occasionally or often discuss sex with their mother. In relation to lifestyle behaviours, about 77% of the young adults have never smoked cigarettes while 15% reported to currently smoke. Most of the participants (56%) also reported having never consumed alcohol and about three quarter (75%) reported to have never used hard substances or drugs.

**Table 1:** Descriptive profile of young adults in the sample.

Socio-Demographic Characteristics	n = 755
**Age Group**	** **
Adolescents (16–19)	207 (27.4%)
Young adults (20–34)	548 (72.6%)
**Sex:**	** **
Female	370 (49.0%)
Male	385 (51.0%)
**Education:**	** **
100 L	229 (30.3%)
200 L	184 (24.4%)
300 L	135 (17.9%)
400+ L	207 (27.4%)
**Family Support**	** **
Receive No Support	58 (7.7%)
Receive Support	697 (92.3%)
**Sexual Experience**	** **
Never Had Sex	141 (18.7%)
Had Sex	614 (81.3%)
**Currently Smoke:**	** **
Never Smoked	582 (77.1%)
No longer Smoke	63 (8.3%)
Currently Smokes	110 (14.6%)
**Currently Drink**	** **
Never Drank Alcohol	425 (56.3%)
No longer Drink	92 (12.2%)
Currently Drinks	238 (31.5%)
**Currently use drug**	** **
Never used Drugs	568 (75.2%)
No longer use Drugs	54 (7.2%)
Currently use Drugs	133 (17.6%)
**Recruitment Site:**	** **
Ilorin	359 (47.6%)
Nasarawa	396 (52.4%)
**Importance of Religion**	
Not Important	21 (2.8%)
Moderately Important	146 (19.3%)
Very Important	588 (77.9%)
**Sex Talk with Dad:**	** **
Never	633 (83.8%)
Occasionally/Often	122 (16.6%)
**Sex Talk with Mum:**	** **
Never	399 (52.8%)
Occasionally/Often	356 (47.2%)

Summary item descriptions of the manifest variables in this study are presented in Table 2. Mean response score for the items ranged from 1.93 for an item measure of self-efficacy for condom purchase and use to 2.42 for an item measure of partner communication self-efficacy for condom use. Results from Table 2 also show the computed correlations between the manifest variables. Among the item measures of partner communication self-efficacy, we observed a statistically significant positive relationship. A similar pattern of relationship was also observed among the item measures of self-efficacy for condom purchase and use.

**Table 2:** Means and correlations of self-efficacy item measures.

		Range	Mean *(SD)*	(1)	(2)	(3)	(4)	(5)	(6)	(7)	(8)	(9)	(10)	(11)
Partner Communication Self-Efficacy														
(1)	partner_eff_05	1–3	2.22 (0.84)	-										
(2)	partner_ eff_06	1–3	2.26 (0.82)	0.718*	-									
(3)	partner_ eff_07	1–3	2.32 (0.82)	0.522*	0.597*	-								
(4)	partner_ eff_08	1–3	2.42 (0.76)	0.447*	0.548*	0.624*	-							
(5)	partner_ eff_09	1–3	2.28 (0.81)	0.378*	0.462*	0.534*	0.583*	-						
(6)	partner_ eff_10	1–3	2.29 (0.80)	0.396*	0.443*	0.483*	0.540*	0.531*	-					
Self-Efficacy for Condom Purchase and Use														
(7)	own_eff_01	1–3	2.09 (0.85)	0.041	0.118*	0.141*	0.128*	0.037	0.042	-				
(8)	own_eff_02	1–3	1.98 (0.88)	0.015	0.076*	0.121*	0.129*	0.056	0.025	0.694*	-			
(9)	own_eff_03	1–3	1.93 (0.88)	-0.032	0.082*	0.127*	0.098*	0.037	0.012	0.674*	0.726*	-		
(10)	own_eff_04	1–3	2.00 (0.89)	0.004	0.089*	0.140*	0.120*	0.065	0.049	0.693*	0.722*	0.714*	-	
(11)	own_eff_12	1–3	2.06 (0.82)	-0.038	0.031	0.097*	0.056	0.009	-0.054	0.492*	0.518*	0.521*	0.502*	-

Note

* p < 0.05

### Measurement model evaluation and results

The measurement model comprised of three latent variables comprising of lifestyle behaviours, partner communication self-efficacy and self-efficacy for condom purchase and use. The results of the evaluation of the model are presented in Table 3. The result shows that all constructs exhibited composite reliability (CR) and internal consistency greater than the acceptable level of 0.70. We observed a high loading of the items on their underlying construct and lower loadings on unrelated constructs. The average variance extracted (AVE) for all the constructs was also above 0.5. This further provided evidence in support of an acceptable convergent validity among the latent variables.

**Table 3:** Summary indicator description and construct reliability.

Construct	Item	Question/indicator	Factor loadings (EFA)	CR	AVE
Partner communication efficacy	Partner_eff_05	I would not feel confident suggesting using condoms with a new partner because I would be afraid, he or she would think I have a sexually transmitted disease	0.649	0.864	0.562
	Partner_eff_06	I would not feel confident suggesting using condoms with a new partner because I would be afraid, he or she would think I thought they had a sexually transmitted disease	0.729		
	Partner_eff_07	I would not feel confident suggesting using condoms with a new partner because I would too shy to	0.813		
	Partner_eff_08	If I were to suggest using a condom to a partner, I would feel afraid that he or she would reject me	0.842		
	Partner_eff_09	If I were unsure of my partner’s feelings about using condoms, I would not suggest using one	0.746		
	Partner_eff_10	If my partner and I were to try to use a condom and did not succeed, I would feel embarrassed to try to use one again (e.g. not being able to unroll condom, putting it on backwards or awkwardness)	0.703		
Self-Efficacy for condom purchase and use	Own_eff_01	I feel confident in my ability to put a condom on myself or my partner	0.813	0.898	0.647
	Own_eff_02	I feel confident I could purchase condoms without feeling embarrassed	0.869		
	Own_eff_03	I feel confident I could remember to carry a condom with me should I need one	0.854		
	Own_eff_04	I feel confident I could gracefully remove and dispose of a condom after sexual intercourse	0.857		
	Own_eff_12	I feel that I know how to use a condom properly.	0.593		
Lifestyle Behaviour	Smoker	Do you currently smoke?	0.858	0.825	0.630
	Drinker	Do you currently smoke?	0.700		
	Drug User	Do you currently use substance/drugs like codeine, smoke weed, tramadol for pleasure or to ease tension?	0.815		

NOTE: All factor loadings are statistically significant at p< 0.05; RMSEA = 0.032 (CI = 0.023–0.041); CFI = 0.974; TLI = 0.967; SRMR = 0.47

Furthermore, we tested the discriminant validity of the constructs by examining how different each one is from other constructs. We found that the factors were also significantly correlated but not very high with a correlation of less than 0.3, which suggests that each latent variable is distinct. An assessment of the difference in the variance shared between each of the constructs and their measures compared to the variance shared between each of the constructs and other measures provided substantial evidence in support of an acceptable discriminant validity [54]. Finally, results of the three-factor confirmatory factor analysis showing the fit statistics as presented in Table 3 support the appropriateness and generalizability of the measurement portion of the model.

### Predictors of condom use efficacy

**Fig 2:**
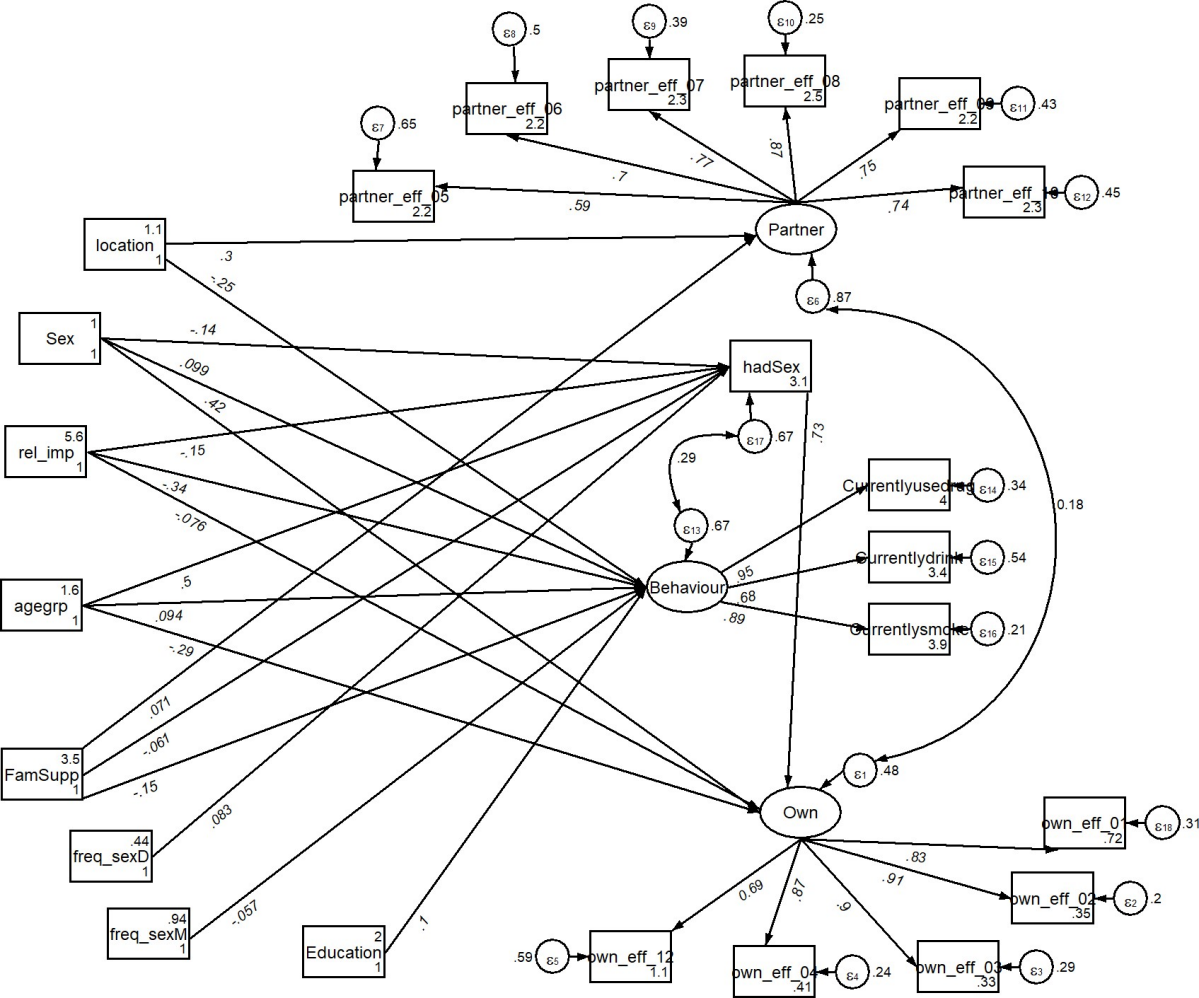
A standardized path model for understanding the predictors of condom use self-efficacy among university students in Nigeria. Note: FamSupp = family support; rel_imp = importance of religious activities; Behaviour = lifestyle behaviours; hadSex = sexual experience. Standardized path coefficients are reported. Statistically insignificant paths are excluded. Error co-variances are also excluded.

We tested our main standardized path coefficients between constructs. The standardized hypotheses of what factors predict self-efficacy for condom use among young adults by analysing coefficients for direct paths with standard error are described in Table 4 and Fig 2. The model fit statistics (RMSEA = 0.020; CFI = 0.988; TLI = 0.982; SRMR = 0.067) provided enough evidence that model fits reasonably for the data. The results from the Bentler-Raykov squared multiple correlations also showed that the model explained slightly more than half of the variance in self-efficacy for condom purchase and use but 13% of the variance in partner communication efficacy.

**Table 4:** Standardized path coefficients of the predictors of condom use self-efficacy.

	Lifestyle Behaviours	Sexual Behaviours	Self- Efficacy for condom purchase and use	Partner Communication Efficacy
	Standardized Coefficients [Standard Error]			
**Endogenous Variables**				
Sexual Experience: *Had Sex*			0.73*** [0.09]	0.08 [0.07]
Lifestyle Behavioural			-0.06 [0.06]	-0.10 [0.05]
**Exogenous Variables**				
Sex: *Male*	0.10*** [0.03]	-0.14** [0.04]	0.42*** [0.04]	0.02 [0.04]
Age Group: *20–34 years*	0.09** [0.03]	0.50*** [0.06]	-0.29*** [0.07]	-0.07 [0.05]
Education	0.10** [0.03]	0.03 [0.05]	0.05 [0.04]	0.01 [0.04]
Family Support: *Received Support*	-0.15*** [0.03]	-0.06** [0.02]	0.04 [0.03]	0.07** [0.03]
Importance of Religion	-0.34*** [0.03]	-0.15*** [0.03]	-0.08* [0.04]	-0.03 [0.03]
Sex Education with Father: *Yes*	0.04 [0.03]	0.08* [0.04]	-0.01 [0.04]	0.01 [0.03]
Sex Education with Mother: *Yes*	-0.06* [0.03]	0.08 [0.04]	0.01 [0.04]	0.04 [0.04]
Recruitment Site: *Nasarawa*	-0.25*** [0.03]	0.06 [0.05]	0.01 [0.04]	0.30*** [0.04]
*Bentler-Raykov squared multiple correlation*	0.332	0.330	0.516	0.128

RMSEA = 0.020 [90CI: 0.010–0.028]; CFI = 0.988; TLI = 0.982; *pclose* = 1.000; SRMR = 0.067

Note

*** p < 0.001

** p < 0.01

* p < 0.05

As shown in Table 4, having some sexual experience was associated with higher self-efficacy for condom purchase and use (β = 0.73, *p<0*.*001*) but not partner communication efficacy (β = 0.08, *p>0*.*05*). Engaging in lifestyle behaviours, on the other hand, was not significantly associated with both self-efficacy for condom purchase and use (β = -0.06, *p>0*.*05*) and partner communication efficacy (β = -0.10, *p>0*.*05*). Demographic characteristics such as age (β = 0.42, *p<0*.*001*) and sex (β = -0.29, *p<0*.*001*) as well as religious importance (β = -0.08, *p<0*.*05*) were directly associated with self-efficacy for condom purchase and use. Receipt of family support (β = 0.07, *p<0*.*01*) and studying in Nasarawa (β = 0.30, *p<0*.*001*) on the other hand were directly associated with higher partner communication efficacy.

To further understand the pathways through which socio-demographic, parental and behavioural factors affect condom use self-efficacy, we present the result of the total, direct and indirect relationships in Table 5. Sexual experience had the largest direct and total effect on self-efficacy for condom purchase and use (β = 0.73, *p<0*.*001*). Among the sociodemographic and parental factors, the results show that sex had the largest total effect on condom purchase and use (β = 0.31, *p<0*.*001*) while the dummy indicator of recruitment site (β = 0.33, *p<0*.*001*) and the receipt of family support (β = 0.08, *p<0*.*01*) had larger significant total effect on partner communication efficacy. The total indirect path from age was the strongest on self-efficacy for condom purchase and use (β = 0.36, p<0.001) but no statistically significant indirect path was observed for partner communication efficacy. In general, sexual experience partially mediated the pathway linking sex, age and the receipt of sex education from father to self-efficacy for condom purchase and use. Sexual experience also mediated the path linking the receipt of family support as well as ratings on religious importance to self-efficacy for condom purchase and use.

**Table 5:** Summary of standardized direct and indirect effects of socio-demographic, parental behavioural factors on condom use self-efficacy.

		Self-efficacy for condom purchase and use		Partner communication efficacy	
		Std. Coeff	SE	Std. Coeff	SE
Sexual Experience: *Had Sex*					
	Total Effect	0.73***	0.39	0.08	0.13
	Direct Effect	0.73***	0.39	0.08	0.13
Lifestyle Behavioural					
	Total Effect	-0.06	0.07	-0.10	0.04
	Direct Effect	-0.06	0.07	-0.10	0.04
Sex: *Male*					
	Total Effect	0.31***	0.05	-0.01	0.03
	Indirect Effect	-0.11**	0.07	-0.02	0.01
	Direct Effect	0.42***	0.07	0.02	0.04
Age Group: *20–34 years*					
	Total Effect	0.08*	0.06	-0.04	0.04
	Indirect Effect	0.36***	0.13	0.03	0.04
	Direct Effect	-0.29***	0.13	-0.07	0.06
Education					
	Total Effect	0.07	0.02	-0.01	0.02
	Indirect Effect	0.02	0.02	-0.01	0.00
	Direct Effect	0.05	0.03	-0.01	0.02
Family Support: *Received Support*					
	Total Effect	0.01	0.08	0.08**	0.05
	Indirect Effect	-0.04*	0.05	0.01	0.01
	Direct Effect	0.04	0.08	0.07**	0.05
Importance of Religion					
	Total Effect	-0.17***	0.05	-0.01	0.03
	Indirect Effect	-0.09**	0.05	0.02	0.02
	Direct Effect	-0.08*	0.06	-0.03	0.03
Sex Education with Father: *Yes*					
	Total Effect	0.05	0.06	0.01	0.04
	Indirect Effect	0.06	0.07	0.00	0.01
	Direct Effect	-0.01	0.08	0.01	0.04
Sex Education with Mother: *Yes*					
	Total Effect	0.04	0.05	0.05	0.03
	Indirect Effect	0.06	0.05	0.01	0.01
	Direct Effect	-0.01	0.06	0.04	0.03
Recruitment Site: *Nasarawa*					
	Total Effect	0.06*	0.05	0.33***	0.04
	Indirect Effect	0.06	0.06	0.03	0.02
	Direct Effect	0.01	0.07	0.30***	0.04

Note

*** p < 0.001

** p < 0.01

* p < 0.05

## Discussion

By examining the predictors of condom use self-efficacy among university students in Nigeria, the present study contributes to the growing body of knowledge on the sexual behaviours of young adults in sub-Saharan Africa. The results of measurement models (confirmatory factor analysis) suggest that the 11 question items that constitute the two latent constructs of self-efficacy appear to be a sound tool for the assessment of self-efficacy for condom use among young people. We also found some consistencies between our proposed model for predicting the determinants of condom self-efficacy and the findings of our studies. The analysis of our structural model showed that behavioural factors had significant direct effects on self-efficacy for condom purchase and use (except lifestyle behaviours) as well as partner communication efficacy, but there were also important roles for demographic and social factors, such as age, sex and parental factors. These findings demonstrate that multidimensional factors, including demographic and behavioural factors, explaining self-efficacy for condom use among young adults.

The result of our analysis highlights that sexual behaviour was the strongest predictor of self-efficacy for condom purchase and use. This finding confirms our assertion that self-efficacy is developed and strengthened through personal experience. Bandura asserts that performance accomplishment is among the sources of self-efficacy [10–12]. Performance accomplishment refers to learning from previous personal experience [10–12]. It is through personal experience that one gets to achieve mastery [10–12]. Young adults who have ever had sex are more likely to have negotiated condom use and used condoms. As a result, the experience gained during this process could have strengthened their condom use self-efficacy.

We had hypothesised that the behavioural factors might also be directly associated with self-efficacy for condom use and purchase as well as partner communication efficacy. We expected that alcohol and drug use might boost confidence or enhance partner communication regarding the use of condoms, it could also limit an individual’s agency to negotiate or use condoms since alcohol intoxication decreases intentions both to negotiate condom use and to use condoms in the future [34, 55]. Our findings, however, provided no evidence in support of this assertion. Also, we observed that the level of partner communication does not differ by both sexual experience and lifestyle factors.

We also observed that the strongest total indirect path to self-efficacy for condom purchase and use was from age and sex. Our model indicates that demographic factors such as age and sex are directly and indirectly related to self-efficacy for condom purchase and use. Both demographic factors were also directly related to sexual experience. Specifically, being a male was negatively associated with sexual experience. Sexually experience, on the other hand, was directly and positively associated with self-efficacy for condom purchase and use, and this finding is consistent with previous studies [28, 56, 57]. Farmer et al. [28] argues that males tend to have higher behavioural control on the use of condoms. The sex differences in condom self-efficacy could be attributable to gender roles and power imbalance in relationships [58, 59]. Gender role and power imbalances are associated with patriarchal, which is the dominant culture in sub-Saharan Africa. The traditional gender role, although changing, disproportionately vested power to men in the sub-Saharan Africa society. Having men decide on the reproductive health of women, permissible under the traditional patriarchy, makes women vulnerable. It is important to note, however, that although patriarchal remains the dominant culture in sub-Saharan Africa; more women are now empowered due to increasing education, modernisation and globalisation. Studies have shown that women in power-balanced relationships tend to exert control over condom use [60, 61].

Interestingly, we observed a negative direct relationship between age and self-efficacy for condom purchase and use, while the total indirect paths had opposite effects. We expected that young adults’ confidence in their ability to use condoms would increase as age increases based on personal learning [28, 45, 62]. Age emerged to be positively related to sexual experience, which in turn led to increased self-efficacy for condom use and purchase. Fortunately, the mediation effect associated with sexual experience was large enough to increase self-efficacy for condom use and purchase.

We also observed a significant indirect effect from ratings of the importance of religion to self-efficacy for condom purchase and use via sexual experience. Young adults who rate religion to be very important in their life were less likely to have had sex and subsequently have low self-efficacy for condom use and purchase. Existing studies have shown that religiosity has a strong positive influence on sexual behaviour and experience of adolescents and young adults [37, 38]. Highly religious young adults in Nigeria are more likely to abstain from sexual activity than less-religious young adults [38]. It is plausible that religious teachings, which emphasise sexual abstinence and preclude the use of contraceptives, could inhibit the condom self-efficacy of young adults.

Among the parental factors, the paths linking the receipt of family support and sex education from father to self-efficacy for condom purchase and use were mediated by sexual experience. Young adults who received support from their family were significantly less likely to have had sex which contributed to lower self-efficacy for condom purchase and use while the direct effect was in the opposite direction and of the same effect size. This finding is consistent with another, which shows that parental support is an important predictor of adolescents and young adults’ sexual behaviour [42]. Parental support could persuade young adults to avoid premarital unplanned pregnancy and equip themselves with condom negotiation skills. A previous study has shown that adolescents and young adults believed that unplanned pregnancy would make them lose their parents’ support [63]. Reception of sex education from young adult’s father was also partially mediated by sexual experience but in the direction not expected. Young adults who reported that they discuss sex-related issues with their father were more likely to have had sex which also contributed to higher self-efficacy for condom purchase and use. The mediation path via lifestyle behaviours confirmed that young adults who received sex education from their mother were less likely to engage in lifestyle behaviours which subsequently contributed to higher partner communication efficacy. This finding is consistent with Ritchwood et al.’s [27] study, which concluded that condom use self-efficacy was predicted by parental-teen communication about sex and actual parent-teen communication about sex and dating. Parents are key to promoting the sexual health of youths through increasing contraceptive use, delaying sexual debuts and improving their self-efficacy [39–41].

There are few caveats to the findings of our study. Due to the cross-sectional nature of our survey design, causal inferences may not be implied. Despite the strength of the SEM and its applicability to our data, the direction of causation cannot be ascertained (e.g. sexual inexperienced young adults may be more open in discussing sexual health education with their parents). However, the hypothesized direction is supported by several studies and descriptive findings. Another potential limitation of our study has to do with the adopted methodology that is based on self-reporting of socio-demographic characteristics and measures of self-efficacy, as a result, we cannot rule out the possibility of social desirability bias in the responses of our participants. Finally, the results of this study can only apply to young adults who are currently enrolled in higher institutions of learning. The omission of young adults who are not in the university or not educated from our study is a serious gap given the potential difference in the needs of this group. This is also likely to limit the generalizability of our study findings. Also, we did not examine whether parents’ wealth status has an effect on self-efficacy for condom use and purchase; thus, future studies could explore this link.

Despite these limitations, it is worthy of note that this study is one of the few studies that attempt to understand the multifaceted factors that predict condom use self-efficacy among university students. By using a structural equation modelling framework, we were able to estimate the covariances between the two dimensions of self-efficacy for condom use simultaneously. We hope that several others will follow our study to identify additional factors related to condom use self-efficacy using a robust and representative (educated and uneducated) sample of young adults. Although we included several covariates which we expect will be directly and indirectly related to partner communication efficacy, findings from our model diagnostics showed that the variables included in the model only accounted for 13% of the variance in partner communication efficacy. This could be an area for future studies particularly in identifying other predictor variables or mediation paths that could explain partner communication efficacy to a larger degree.

Overall, our study affirms that demographic factors such as age, and sex, ratings on religious importance and parental factors such as the receipt of family support had direct predictive effects towards both behavioural factors. Sex education with mother and the number of years spent studying at the university also had a direct predictive effect towards lifestyle behaviours but not sexual behaviours. Most of these factors also showed significantly mediated effects through sexual experience to self-efficacy for condom purchase and use but not partner communication efficacy. In this sense, it is not being male or young adults aged 20–34 years or having higher ratings on religious importance alone that increases self-efficacy but other behavioural factor such as sexual experience through which these factors affect self-efficacy for condom use. These findings have implications for the design of interventions to enhance condom use self-efficacy in Nigeria and beyond. Such interventions need to address and strengthen condom use self-efficacy among the most at-risk group.

## Supporting information

S1 Appendix Summary of exploratory factor analysis results for Self-efficacy for condom purchase and use, partner communication efficacy, and lifestyle behavioural measures using iterated principal factor analysis estimation (N = 755). (DOCX)

S1 Dataset Dataset for understanding the predictors of condom use self-efficacy among university students in Nigeria. (XLSX)
