# Supplementary material for: What predicts self-efficacy? Understanding the role of sociodemographic, behavioural and parental factors on condom use self-efficacy among university students in Nigeria
Source: PLoS One. 2019 Aug 28;14(8):e0221804. doi: 10.1371/journal.pone.0221804 (PMC6713390; doi:10.1371/journal.pone.0221804)
Supplement: S1 Appendix — (DOCX) [file pone.0221804.s001.docx]

Appendix 1: Summary of exploratory factor analysis results for self-efficacy and lifestyle behavioural measures using iterated principal factor analysis estimation (N = 755)

| Variable | Factor Loadings | | | Uniqueness |
| --- | --- | --- | --- | --- |
|  | Self- efficacy for condom purchase and use | Partner communication efficacy | Lifestyle Behaviours |  |
| partner_ eff_05 | -0.0325 | **0.6833** | -0.0361 | 0.5307 |
| partner_ eff_06 | 0.0584 | **0.7765** | -0.022 | 0.3932 |
| partner_ eff_07 | 0.1234 | **0.7708** | -0.0335 | 0.3895 |
| partner_ eff_08 | 0.1005 | **0.7604** | -0.0897 | 0.4037 |
| partner_ eff_09 | 0.0304 | **0.6758** | -0.0356 | 0.5412 |
| partner_ eff_10 | -0.0183 | **0.6519** | -0.0227 | 0.5742 |
| own_eff_01 | **0.7913** | 0.0765 | 0.0938 | 0.3592 |
| own_eff_02 | **0.8202** | 0.0545 | 0.0983 | 0.3147 |
| own_eff_03 | **0.8145** | 0.0343 | 0.1209 | 0.3207 |
| own_eff_04 | **0.8247** | 0.0638 | 0.0798 | 0.3094 |
| own_eff_11 | 0.1641 | -0.0236 | -0.1279 | 0.9562 |
| own_eff_12 | **0.6708** | -0.0165 | 0.0086 | 0.5497 |
| own_eff_13 | 0.4307 | 0.0441 | -0.1178 | 0.7987 |
| currently_smoke | 0.1556 | -0.0987 | **0.8083** | 0.3128 |
| currently_drink | 0.3075 | -0.0019 | **0.6215** | 0.5192 |
| currently_drug | 0.1397 | -0.086 | **0.788** | 0.3522 |
| Variance Explained | 3.4742 | 3.1578 | 1.7429 |  |
| Proportion of Variance Explained | 0.4148 | 0.3771 | 0.2081 |  |
